# Target Cell Availability, Rather than Breast Milk Factors, Dictates Mother-to-Infant Transmission of SIV in Sooty Mangabeys and Rhesus Macaques

**DOI:** 10.1371/journal.ppat.1003958

**Published:** 2014-03-06

**Authors:** Ann Chahroudi, Emily Cartwright, S. Thera Lee, Maud Mavigner, Diane G. Carnathan, Benton Lawson, Paul M. Carnathan, Tayebeh Hashempoor, Megan K. Murphy, Tracy Meeker, Stephanie Ehnert, Christopher Souder, James G. Else, Joyce Cohen, Ronald G. Collman, Thomas H. Vanderford, Sallie R. Permar, Cynthia A. Derdeyn, Francois Villinger, Guido Silvestri

**Affiliations:** 1 Department of Pediatrics, Emory University School of Medicine, Atlanta, Georgia, United States of America; 2 Yerkes National Primate Research Center, Atlanta, Georgia, United States of America; 3 Departments of Pathology and Laboratory Medicine, Emory University School of Medicine, Atlanta, Georgia, United States of America; 4 University of Pennsylvania Perelman School of Medicine, Philadelphia, Pennsylvania, United States of America; 5 Duke Human Vaccine Institute, Durham, North Carolina, United States of America; University of North Carolina at Chapel Hill, United States of America

## Abstract

Mother-to-infant transmission (MTIT) of HIV is a serious global health concern, with over 300,000 children newly infected in 2011. SIV infection of rhesus macaques (RMs) results in similar rates of MTIT to that of HIV in humans. In contrast, SIV infection of sooty mangabeys (SMs) rarely results in MTIT. The mechanisms underlying protection from MTIT in SMs are unknown. In this study we tested the hypotheses that breast milk factors and/or target cell availability dictate the rate of MTIT in RMs (transmitters) and SMs (non-transmitters). We measured viral loads (cell-free and cell-associated), levels of immune mediators, and the ability to inhibit SIV infection in vitro in milk obtained from lactating RMs and SMs. In addition, we assessed the levels of target cells (CD4+CCR5+ T cells) in gastrointestinal and lymphoid tissues, including those relevant to breastfeeding transmission, as well as peripheral blood from uninfected RM and SM infants. We found that frequently-transmitting RMs did not have higher levels of cell-free or cell-associated viral loads in milk compared to rarely-transmitting SMs. Milk from both RMs and SMs moderately inhibited in vitro SIV infection, and presence of the examined immune mediators in these two species did not readily explain the differential rates of transmission. Importantly, we found that the percentage of CD4+CCR5+ T cells was significantly lower in all tissues in infant SMs as compared to infant RMs despite robust levels of CD4+ T cell proliferation in both species. The difference between the frequently-transmitting RMs and rarely-transmitting SMs was most pronounced in CD4+ memory T cells in the spleen, jejunum, and colon as well as in central and effector memory CD4+ T cells in the peripheral blood. We propose that limited availability of SIV target cells in infant SMs represents a key evolutionary adaptation to reduce the risk of MTIT in SIV-infected SMs.

## Author Summary

Currently 2.5 million children are infected with HIV, largely as a result of mother-to-child transmission, and there is no effective vaccine or cure. Studies of Simian Immunodeficiency Virus (SIV) infection of nonhuman primate species termed “natural hosts” have shown that mother-to-infant transmission of SIV in these animals is rare. Natural hosts are African monkey species that are naturally infected with SIV in the wild but do not develop AIDS. We sought to understand the mechanism by which natural hosts are protected from mother-to-infant transmission of SIV, aiming to translate our findings into novel strategies to prevent perinatal HIV infection. We found that natural host sooty mangabey infants have extremely low levels of target cells for SIV infection in lymphoid and gastrointestinal tissues. Direct comparison of infant sooty mangabeys and infant rhesus macaques (non-natural host species with high SIV transmission rates) confirmed that natural hosts have significantly lower levels of SIV target cells compared with non-natural hosts. Analysis of the breast milk of sooty mangabeys and rhesus macaques revealed similar levels of virus and ability to inhibit SIV infection. Our study provides evidence for target cell restriction as the main mechanism of protection from mother-to-infant SIV transmission in natural hosts.

## Introduction

Worldwide, over 30 million people are infected with HIV, including 3.3 million children. Transmission through breastfeeding can account for almost half of pediatric HIV infections [1]. Recent World Health Organization recommendations support breastfeeding by HIV-infected women along with antiretroviral therapy for mother or infant in areas where alternative feeding is not feasible. However, the high cost of anti-retroviral medications and limited access to therapy in developing countries along with unknown long-term consequences of the use of ART during breastfeeding demand further investigations into novel interventions to prevent breast milk-related transmission of HIV. A major barrier to the development of these interventions is a lack of understanding of the mechanisms that mediate breastfeeding transmission of HIV.

Many species of African nonhuman primates, including the sooty mangabey (*Cercocebus atys*) are naturally infected with simian immunodeficiency virus (SIV). In contrast to SIV infection of Asian macaques and HIV infection of humans (non-natural hosts), natural SIV infections are typically non-pathogenic (reviewed in [2]). These differential outcomes of SIV/HIV infection are consistently observed despite high-level virus replication in both natural and non-natural hosts. We have recently demonstrated that mother to infant transmission (MTIT) is rare in naturally SIV-infected sooty mangabeys (SMs, <7%), compared to the much higher rates seen in SIV-infected rhesus macaques (RMs, 25–75%) and HIV-infected humans (∼40%) [3]–[7]. The difference between natural and non-natural hosts is particularly striking in terms of breastfeeding transmission, given that post-partum experimental SIV infection of lactating mandrills (natural hosts) resulted in no MTIT events compared to the 60–75% transmission rate seen in breastfeeding RMs (non-natural hosts) [4], [6], [8]. Thus, over thousands of years of virus-host interaction, natural SIV hosts have evolved mechanisms to render SIV infection non-pathogenic and to restrict SIV transmission from mothers to infants. The rarity of MTIT in natural hosts has been hypothesized to be due to low levels of CD4+CCR5+ target cells for SIV infection found in peripheral blood and mucosal sites [8], [9]. However, an in depth comparative analysis of target cell availability in multiple tissues had not previously been conducted in nonhuman primates.

In the current study, we investigated several potential mechanisms to explain the differential rates of MTIT in natural and non-natural hosts. Specifically, we asked whether the low rate of MTIT observed in SMs could be due to i) low levels of virus present in breast milk; ii) enhanced inhibitory properties of breast milk, and/or iii) limited availability of SIV target cells in infants. By directly comparing SMs and RMs we found similar properties in the breast milk of these natural and non-natural host nonhuman primate species. However, we observed strikingly lower levels of CD4+CCR5+ SIV target cells in infant SMs as compared to infant RMs. Based on these data we propose that limited target cell availability, rather than breast milk specific factors, is responsible for the rarity of mother-to-infant transmission of SIV in SMs.

## Results

### Pharmacological induction of lactation

To investigate the virologic and immunologic features of breast milk that may influence transmission of SIV in nonhuman primates, we pharmacologically induced lactation in female non-pregnant chronically SIV-infected RMs and SMs. Clinical features of the study animals are listed in Table 1. RMs and SMs were experimentally infected with SIVmac239 and SIVsmm, respectively. Viral loads prior to pharmacologic induction of lactation (during chronic infection) ranged from 5.7×10^3^ to 3.8×10^5^ copies/ml plasma. In keeping with the nonpathogenic nature of SIV infection of SMs, CD4+ T cell counts were higher in SMs than RMs (mean of 450 cells/mm^3^ compared to 227 cells/mm^3^).

**Table 1:** Clinical characteristics of chronically SIV-infected, female nonhuman primates.

	Date of Birth	Date of SIV Infection	SIV Strain	Plasma Viral Load (copies/ml)	CD4 Count (cells/mm^3^)
**Rhesus macaques**					
**RTi12**	8/27/07	3/24/10	SIVmac239	3.6×10^4^	391
**RRw9**	5/17/07	3/31/10	SIVmac239	1.7×10^5^	110
**RZb9**	4/20/02	3/31/10	SIVmac239	3.8×10^5^	180
**Sooty mangabeys**					
**FCs**	5/17/94	10/31/00	SIVsmm	3.9×10^4^	585
**FUq**	7/16/93	10/31/00	SIVsmm	5.7×10^3^	304
**FLn**	5/23/91	5/10/99	SIVsmm	1.9×10^4^	461

The protocol used to induce lactation has previously been tested in RMs and African green monkeys and the breast milk produced is immunologically similar to that of milk collected from naturally-lactating monkeys [10], [11]. This protocol involves treating the animals with medroxyprogesterone and estradiol to mature the mammary glands, haloperidol (a dopamine receptor antagonist) to raise endogenous prolactin levels, and oxytocin given prior to milk expression to simulate the milk ejection reflex. Lactation was successfully induced in all animals by weeks 4–6 and was sustained for 5 months. Throughout the lactation period, we monitored for effects of the used treatments on mononuclear cells in peripheral blood. Specifically, we measured the relative percentage of mononuclear cell subsets, the differentiation status of CD4+ and CD8+ T cells, as well as the level of T cell activation by flow cytometry. Minor perturbations were seen at various time points, but the pharmacologic agents did not induce a sustained change in any of the measured parameters (data not shown).

### Similar level of cell-free and cell-associated SIV in breast milk from RMs and SMs

**Figure 1:**
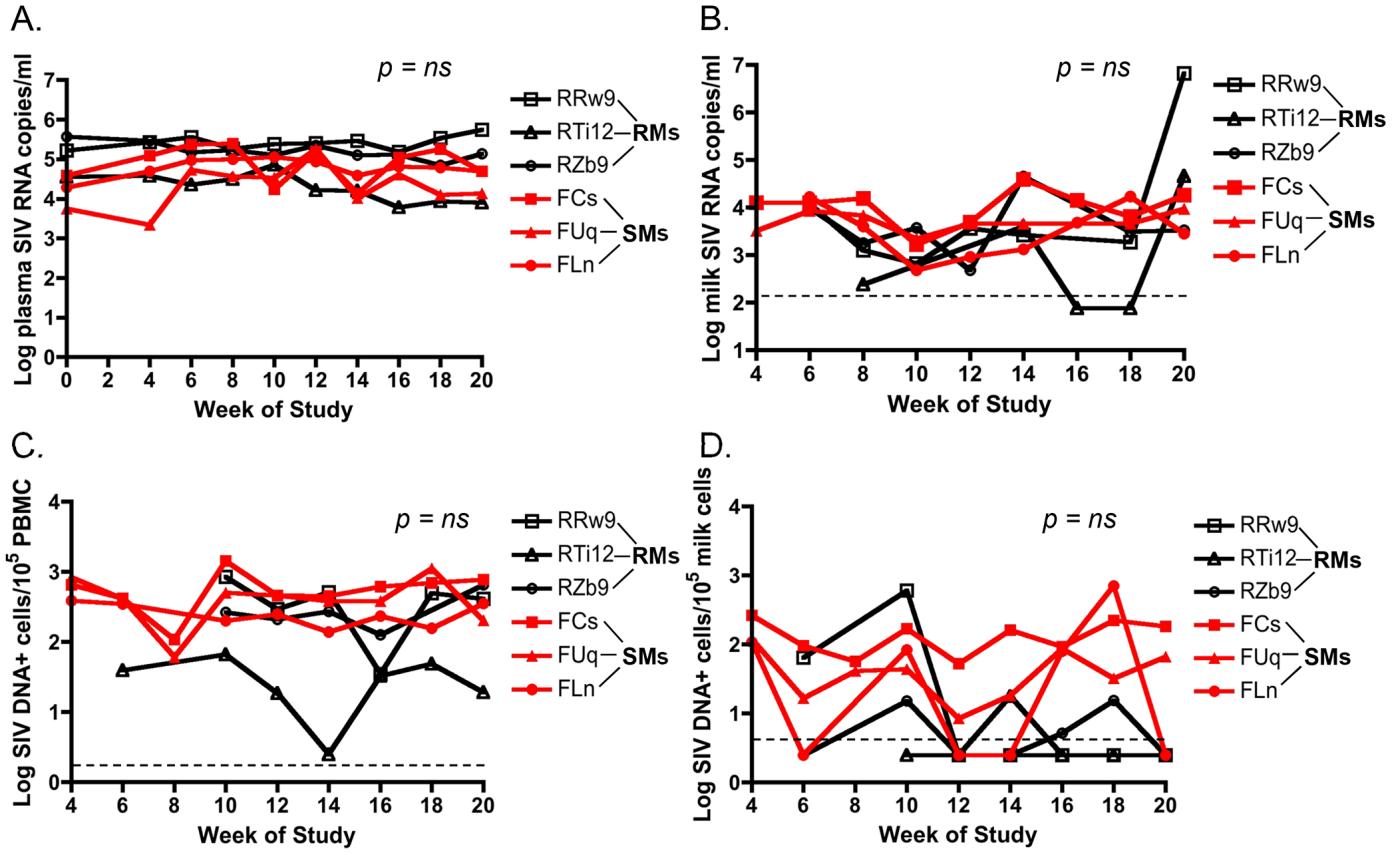
Similar cell-free and cell-associated SIV levels in blood and milk from RMs and SMs following pharmacologic induction of lactation. Lactation was induced using intramuscular injections of estradiol and medroxyprogesterone along with oral haloperidol followed by oxytocin injections to stimulate the milk ejection reflex. Over the 20 weeks of this study, plasma viral loads were measured via SIV RNA real time RT-PCR every 2 weeks (A). Breast milk was collected from RMs and SMs and cell-free SIV RNA was quantified in the de-fatted milk fraction by RT-PCR (B). Cell-associated SIV DNA was then measured in both PBMCs (C) and breast milk cells (D) with simultaneous albumin detection to determine cell number per reaction. Dashed lines represented the lower limit of detection for the assay. Undetectable values were plotted as half the lower limit of detection. Area under the curve analyses followed by nonparametric Mann-Whitney test was used to assess significance.

The level of HIV in plasma and breast milk of HIV-infected women correlates with mother to child transmission (MTCT) of HIV [12]–[17]. To investigate the viral loads in the milk of SMs and RMs undergoing hormone-induced lactation we first measured the level of cell-free SIV RNA by RT-PCR in de-fatted milk as well as plasma from these animals. As shown in Figure 1A, we found that plasma viremia was stable over the period of lactation and was similar in both RMs and SMs. Figure 1B shows that there was no overall difference in the level of cell-free SIV RNA in milk from RMs and SMs. At week 20, temporally associated with a case of clinical mastitis, the macaque RRw9 did exhibit an increased level of SIV RNA in milk compared to other animals. We next measured cell-associated SIV DNA in breast milk cells and PBMCs from RMs and SMs, as several researchers have proposed that the level of cell-associated HIV, rather than cell-free HIV, is the main mediator of MTCT via breast milk [12], [15], [16]. We were able to detect SIV DNA in PBMCs from both RMs and SMs, but levels were more variable in RMs (Fig. 1C). Interestingly, SIV DNA was infrequently detected in milk cells from RMs, but was more consistently detected in milk cells from SMs (Fig. 1D). For each virologic comparison, area under the curve analysis followed by the nonparametric Mann-Whitney test showed no significant difference between cell-free or cell-associated viral loads in RMs vs. SMs. Taken together, these comparative data on virus load in the milk of RMs and SMs do not support the hypothesis that lower virus levels (cell-free or cell-associated) are responsible for reduced SIV transmission to infant SMs.

### T cell differentiation state does not differ in milk cells of RMs and SMs

**Figure 2:**
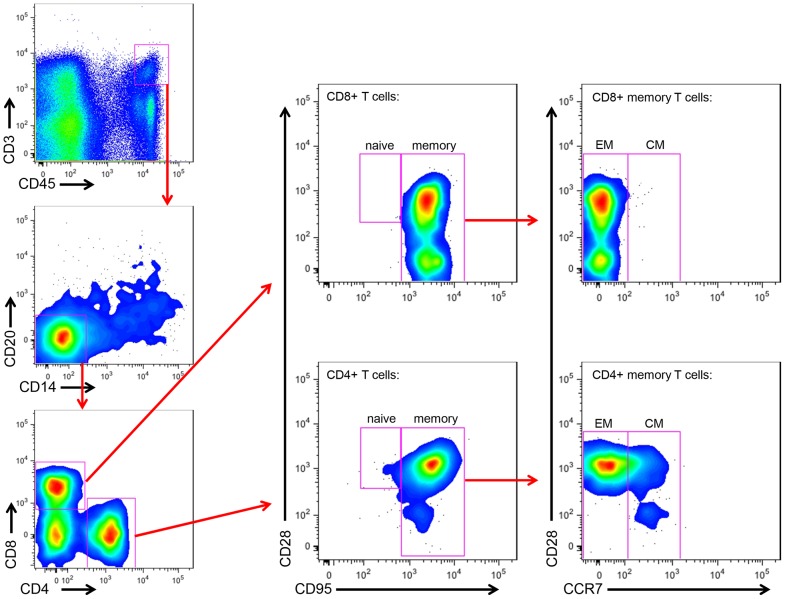
Memory and effector CD4+ and CD8+ T cell subsets in SM milk. The cellular fraction of breast milk was stained with monoclonal antibodies to identify the relative proportions of T cell subsets. Gating strategy was as follows: CD45 vs CD3 to identify hematopoietic T cells, then CD20 vs CD14 to exclude B cells and monocytes/macrophages, respectively, then CD4 vs CD8. The CD4+ and CD8+ populations were stained with CD28 and CD95 to identify naïve (CD28+CD95−) and memory (CD28+/−CD95+) subsets. The memory population was further differentiated into effector (CCR7−) and central (CCR7+) memory subsets.

As we found that the viral loads in the milk did not differ between RMs and SMs, we next sought to determine whether the immunologic features of milk might account for the differential rates of MTIT of SIV in these two species. To this end, we analyzed the relative proportions of CD4+ and CD8+ T cells and their subsets in breast milk from RMs and SMs (see Fig. 2 for representative flow plots). CD45 was used to distinguish cells of hematopoietic origin in milk. We found that CD4+ T cells comprised 19.9–36.2% of CD45+CD3+ live cells in SMs and 1.7–22.9% in RMs (Table 2). We next measured the T cell differentiation status of CD4+ and CD8+ T cells derived from RM and SM milk. As expected, the vast majority of CD4+ and CD8+ T cells in milk from SMs and RMs possessed a memory phenotype (>93%, Table 2). Memory CD4+ T cells from SMs were predominantly comprised of effector memory cells (CD28+/−CD95+CCR7−), consistent with the possibility that these cells are the major contributors to the observed level of cell-associated SIV DNA in milk.

**Table 2:** T cell subsets in milk from RMs and SMs.

	% CD45+CD3+ CD4+ T cells	% Naïve^1^ CD4+ T cells	% Total Memory^2^ CD4+ T cells	% Central Memory^3^ CD4+ T cells	% Effector Memory^4^ CD4+ T cells
**Rhesus macaques**					
**RTi12**	22.9	3.1	93.5	63.0	37.1
**RRw9**	18.7	*	*	*	*
**RZb9**	1.7	2.4	97.6	18.5	81.1
**Sooty mangabeys**					
**FCs**	19.9	0.4	98.2	15.0	82.7
**FUq**	36.2	1.1	97.9	20.2	79.7
**FLn**	22.5	3.4	95.0	24.0	75.9

	% CD45+CD3+ CD8+ T cells	% Naïve^1^ CD8+ T cells	% Total Memory^2^ CD8+ T cells	% Central Memory^3^ CD8+ T cells	% Effector Memory^4^ CD8+ T cells
**Rhesus macaques**					
**RTi12**	14.0	0.1	99.9	2.4	97.6
**RRw9**	53.1	6.5	93.5	2.3	97.7
**RZb9**	87.6	2.7	97.3	2.1	97.9
**Sooty mangabeys**					
**FCs**	40.8	0.1	99.7	0.4	98.3
**FUq**	31.4	0.1	99.7	0.9	98.6
**FLn**	28.8	1.9	95.2	2.3	97.7

1 Naïve  =  CD45+CD3+CD28+CD95−.

2 Memory  =  CD45+CD3+CD28+/−CD95+.

3 Central Memory  =  CD45+CD3+CD28+/−CD95+CCR7+.

4 Effector Memory  =  CD45+CD3+CD28+/−CD95+CCR7−.

*insufficient cells.

### Similar level of immune mediators in breast milk from RMs and SMs

To further investigate the immunologic profile of milk, we measured the level of cytokines and chemokines in de-fatted milk from SIV-infected RMs and SMs following pharmacologic induction of lactation. We found that all of the tested immunological factors were present at similar levels in SIV-infected RMs and SMs (Table 3, left columns). Although not statistically significant (likely related to sample size), milk from SIV-infected RMs contained higher levels of RANTES and IL-15 compared to milk from SIV-infected SMs. Higher milk levels of IL-15 have been associated with protection from HIV MTCT [18]; in contrast, RANTES in milk has been positively correlated with MTCT of HIV [19], [20], thereby confusing our interpretation of the elevated levels of both of these factors in RMs. We also compared the levels of cytokines/chemokines in milk and plasma within each species and found that, with the exception of IL-12 p40 in RMs and Eotaxin in SMs, milk contains higher levels of all tested immune factors (data not shown).

**Table 3:** Immune mediators in milk (pg/ml) of SIV-infected and uninfected nonhuman primates.

	SIV-infected, induced lactation			Uninfected, naturally lactating			
	Rhesus macaques	Sooty mangabeys	*p value*	Rhesus macaques	Sooty mangabeys	*p value*	
**MIP-1α**	87.6	109.6	*1*	12.6	13.4	*0.2949*	
**MIP-1β**	55.5	80.3	*1*	6.5	5.1	*0.1282*	
**RANTES**	1405.8	397.5	*0.1*	n.d.	n.d.	*n.a.*	
**IL-1Rα**	144.0	381.6	*0.2*	46.3	64.2	*0.1649*	
**IL-1β**	29.6	26.1	*0.7*	18.1	14.8	*<0.05*	
**IL-2**	192.3	244.3	*0.4*	109.9	92.5	*0.053*	
**IL-4**	6.8	13.4	*0.1*	n.d.	n.d.	*n.a.*	
**IL-5**	1.1	0.4	*0.7*	n.d.	n.d.	*n.a.*	
**IL-6**	195.6	182.5	*0.7*	187.6	132.1	*<0.05*	
**IL-8**	n.d.	5234.2	*0.1*	n.d.	58.1	*<0.001*	
**IL-10**	47.0	56.5	*0.7*	43.1	33.2	*<0.01*	
**IL-12p40**	456.0	762.0	*0.4*	276.0	273.5	*0.9015*	
**IL-15**	2029.8	517.5	*0.1*	53.8	27.5	*<0.05*	
**IL-17**	n.d.	n.d.	*n.a.*	n.d.	n.d.	*n.a.*	
**TNF-α**	74.4	102.3	*0.2*	7.2	4.2	*0.0728*	
**GM-CSF**	4.3	6.6	*0.1*	3.2	2.8	*0.1282*	
**G-CSF**	193.3	229.9	*0.7*	96.2	87.8	*0.2593*	
**MDC**	2120.0	10286.9	*0.4*	n.d.	n.d.	*n.a.*	
**MIF**	2941.2	3531.6	*0.7*	833.0	1136.2	*0.1282*	
**MIG**	424.7	3831.8	*0.1*	n.d.	n.d.	*n.a.*	
**MCP-1**	36825.3	87352.4	*0.4*	30.4	99.2	*<0.05*	
**IFN-γ**	28.3	77.2	*0.1*	9.4	8.8	*0.3176*	
**I-TAC**	31547.1	37069.8	*1*	125.7	34.3	*0.053*	
**EGF**	2009.2	511.0	*0.1*	639.3	356.0	*<0.05*	
**HGF**	398.8	2139.8	*0.2*	8.5	5.7	*0.0732*	
**VEGF**	302.1	343.5	*0.7*	335.7	192.4	*<0.05*	
**FGF-basic**	29.1	48.1	*0.1*	19.4	19.3	*0.7104*	
**Eotaxin**	89.1	41.2	*0.4*	2.1	0.7	*<0.05*	

n.d., none detected.

n.a., not applicable.

Recognizing that our sample size of infected animals was small, we further characterized immune mediators in milk of seven naturally-lactating, SIV-uninfected RMs and SMs. We were not able to perform the same comparison among naturally-lactating, SIV-infected animals as infected RMs are excluded from the breeding colony. Milk was collected by manual expression between 65 and 255 days post-partum and the de-fatted milk was used in a multiplex panel. We found that milk from uninfected SMs had elevated IL-8 compared to uninfected RMs (p<0.001, Table 3, right columns); SIV-infected SMs also had higher levels of this chemokine compared to SIV-infected RMs (as well as uninfected SMs), although this comparison was not statistically significant. When statistical significance was assessed at the p<0.01 level, uninfected RMs had higher levels of IL-10 compared to uninfected SMs. At the p<0.05 level, uninfected RMs had higher levels of IL-1β, IL-6, IL-15, EGF, VEGF, and Eotaxin, but a lower level of MCP-1. We did not detect a difference between RMs and SMs in the levels of the CCR5 ligands MIP-1α, MIP-1β, or RANTES in the SIV-uninfected animals (Table 3, right columns). Overall, while some differences were seen between RMs and SMs, a particular pattern that could be linked to enhanced or reduced transmission did not emerge in either the naturally-lactating or pharmacologically-lactating animals.

### Breast milk from RMs and SMs similarly inhibits SIV infection in vitro

**Figure 3:**
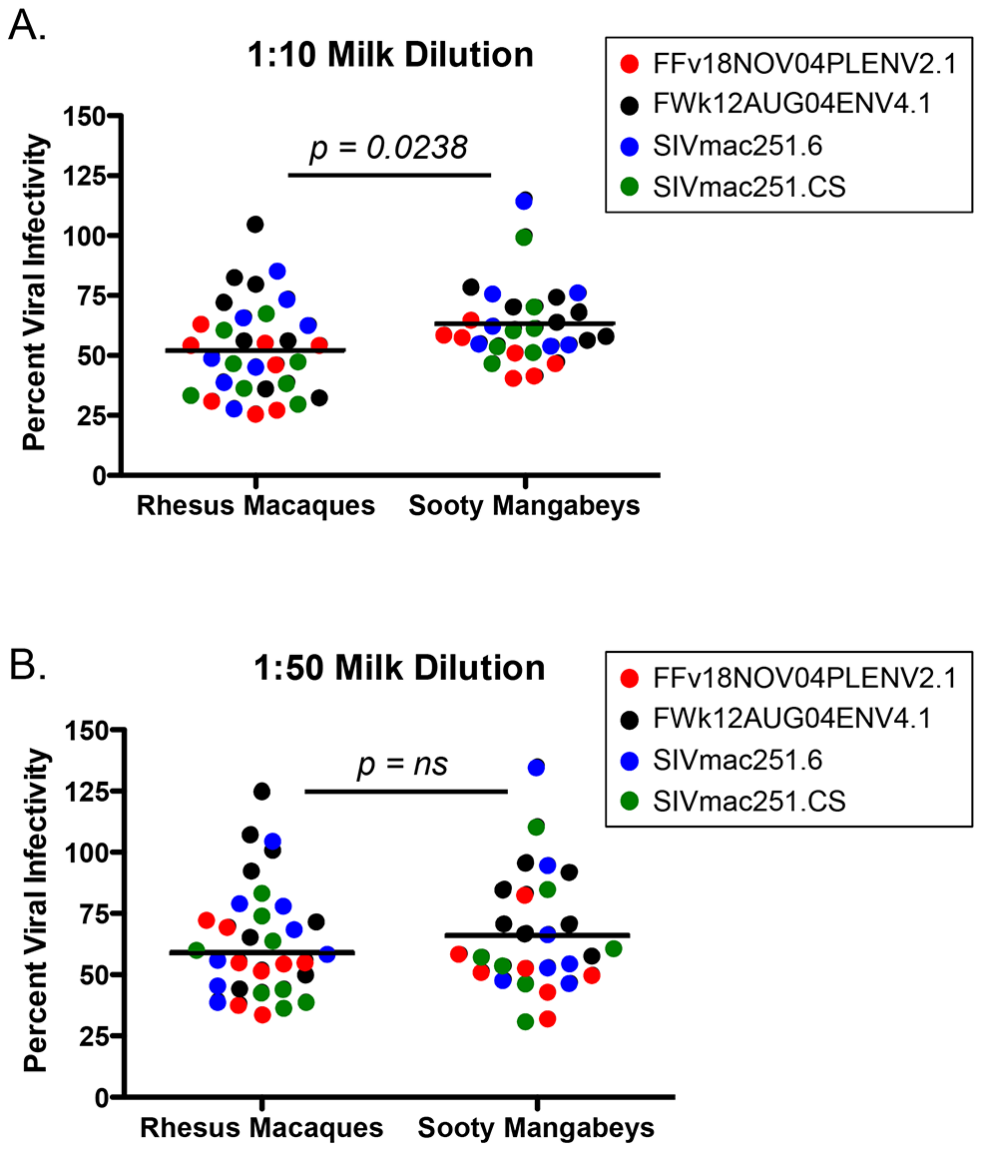
Inhibition of SIV infection by milk from RMs and SMs. The inhibitory capacity of serial dilutions of de-fatted breast milk from eight RMs and seven SMs against SIVsmm subtype 1 (FFv18NOV04PLENV2.1), SIVsmm subtype 2 (FWk12AUG04ENV2.1), SIVmac251.6 (neutralizing antibody-sensitive), or SIVmac251.CS (neutralizing antibody-resistant) viral envelopes was determined in the Tzm-bl neutralization assay. Comparisons between RMs and SMs were made at the dilutions where inhibition was seen. Adjusted against wells including no milk, percent viral infectivity in the presence of 1∶10 diluted (A) or 1∶50 diluted (B) milk is depicted on the vertical axis. Horizontal lines depict the median of each group. A nonparametric Mann-Whitney test was used to determine significance.

Many cross-sectional and in vitro studies using human milk support a role for innate factors in conferring either resistance to or promotion of transmission (reviewed in [21]). Additionally, previous work has identified specific factors in semen (SEVI) or blood (VIRIP) that respectively promote or restrict HIV infection [22], [23]. While we did not find candidate inhibitors (or enhancers) of breastfeeding transmission of SIV in milk from SMs or RMs among the studied cytokines or chemokines, these experiments did not directly rule out the hypothesis that other factors, including innate milk constituents such as lactoferrin or mucin, may directly influence the infectivity of SIV in milk. Therefore, we sought to test directly whether milk from SMs was more able to inhibit in vitro SIV infection compared to milk from RMs. For these studies only milk from naturally-lactating, SIV-uninfected animals was used. Viral pseudotypes expressing envelope (Env) glycoproteins from two SMs naturally infected with SIVsmm subtypes 1 (FFv18NOV04PLENV2.1) and 2 (FWk12AUG04ENV4.1) and neutralizing antibody-sensitive and -resistant SIVmac251 Envs (SIVmac251.6 and SIVmac251.CS, respectively) were used to infect the Tzm-bl reporter cell line. As shown in Figure 3, we found that de-fatted milk diluted 1∶10 and 1∶50 from both SMs and RMs generally inhibited in vitro SIV infection by 40–50%, with RMs exhibiting more efficient inhibition at the 1∶10 dilution (p = 0.0238, Mann-Whitney test). SMs were able to equally inhibit infection of genetically divergent SIVsmm and SIVmac251 Envs, suggesting that this inhibition was not virus strain-specific and potentially not Env-dependent. The same was seen for RM milk inhibition of both the SIVmac251 and SIVsmm Envs. Thus, while we did observe a modest inhibition of in vitro SIV infection in the presence of SM or RM milk, there was no difference in inhibitory capacity between these two species.

### Robust levels of CD4+ T cell proliferation in both SM and RM infants

As we did not find specific differences in the breast milk of frequently-transmitting RMs vs. rarely-transmitting SMs, we next investigated whether these differential rates of MTIT could be better explained by infant factors. Previous work from our group has shown restricted expression of CCR5 on central memory CD4+ T cells from adult SMs [24], and a paucity of CCR5 on circulating CD4+ T cells from infant mandrills was proposed as a mechanism to explain the absence of breastfeeding transmission in this species [8]. Therefore, we next sought to measure the availability of SIV target cells in infant SMs and RMs, focusing on tissues along the gastrointestinal tract as well as lymph nodes, by performing multicolor flow cytometry on cells isolated from nonhuman primate infant tissues at necropsy. Table 4 shows the ages and causes of death of the SM and RM infants whose tissues were studied. All infants were born to SIV negative mothers and, for the SMs, all parents were homozygous wild type for CCR5.

**Table 4:** Clinical features of nonhuman primate infants.

Animal Number	Age at Death (days)	Cause of Death	Dam's SIV Status
**RM1**	0	Euthanized in utero (maternal weight loss).	Negative
**RM2**	1	Neurologic deficits. Euthanized.	Negative
**RM3**	27	Chronic diarrhea. Euthanized.	Negative
**RM4**	112	Chronic diarrhea. Euthanized.	Negative
**RM5**	138	Chronic diarrhea. Euthanized.	Negative
**RM6**	287	Chronic diarrhea. Euthanized.	Negative
**RM7**	312	Chronic diarrhea and pulmonary edema. Euthanized.	Negative
**SM1**	0	Stillbirth.	Negative
**SM2**	0	Prematurity, infanticide.	Negative
**SM3**	1	Maternal abuse. Euthanized.	Negative
**SM4**	4	Prematurity, hypoglycemia, maternal neglect. Euthanized.	Negative
**SM5**	5	Prematurity, presumed sepsis.	Negative
**SM6**	10	Maternal neglect, presumed sepsis. Euthanized.	Negative
**SM7**	15	Maternal abuse, hypoglycemia, respiratory distress. Euthanized.	Negative
**SM8**	107	Neurologic deficits. Euthanized.	Negative

**Figure 4:**
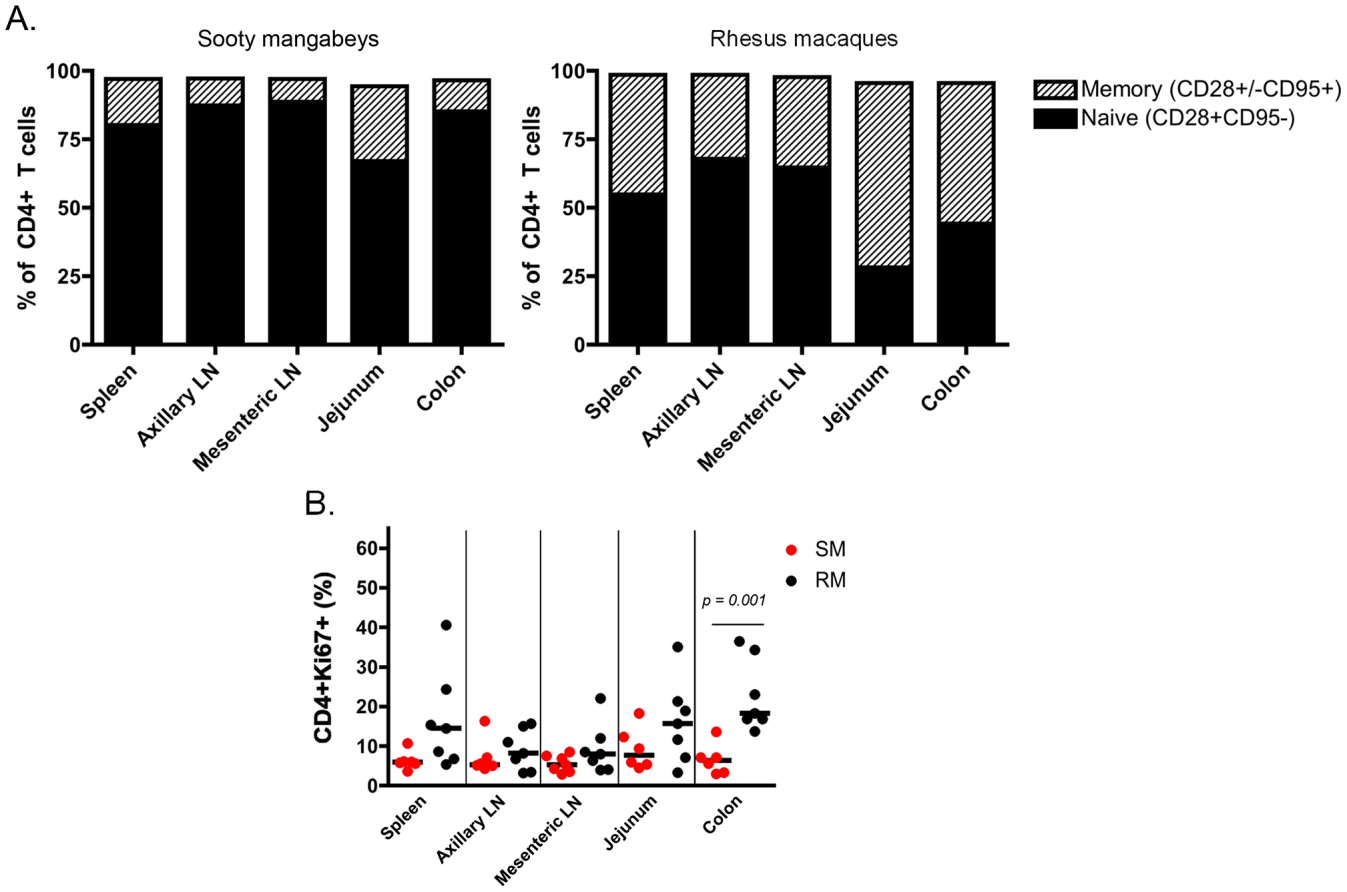
T cell differentiation and proliferation in infant SM and RM tissues. (A) The proportion of naïve (CD28+CD95−) and memory (CD28+/−CD95+) CD4+ T cells in lymphoid and gastrointestinal tissues of infant SMs and RMs was measured at necropsy. For each tissue site, the total may not add to 100% because of minor populations excluded in gating. (B) The level of cell activation/proliferation was measured in CD4+ T cells isolated from multiple tissues of infant RMs and SMs using the marker Ki-67. Mann-Whitney test was used to determine significance. Comparisons without p value shown were non-significant.

It should be noted that this is the first immunological analysis of infant SM blood and tissues. As such, we performed a detailed characterization of the CD4+ T cells obtained. Naïve and memory T cells were defined by expression of CD28 and CD95 as previously described [25]. As expected based on the age of these animals, the majority of CD4+ T cells had a naïve phenotype (Fig. 4A), but a greater percentage of memory CD4+ T cells was found in the upper intestine compared to other sites (mean of 27% in the jejunum). At lymphoid sites, RM infants had a similar predominance of naïve over memory CD4+ T cells, but the gastrointestinal tissues and spleen of RMs had an increased proportion of memory CD4+ T cells compared to the same sites in SMs (Fig. 4A), a finding that could be due in part to the slightly older age of the RM infants available for study. We then measured the percentage of proliferating cells in infant RMs and SMs at these tissue sites using the marker Ki-67. We found robust levels of proliferation in CD4+ T cells from both RMs and SMs (SMs: range 3–18% Ki-67+; RMs: range 3–40% Ki-67+) (Fig. 4B). These levels of CD4+ T cell proliferation are higher than previously seen in adult SMs [26]–[28] and are likely due to the developing infant immune system.

### Limited CCR5 expression on CD4+ T cells from SM infants

**Figure 5:**
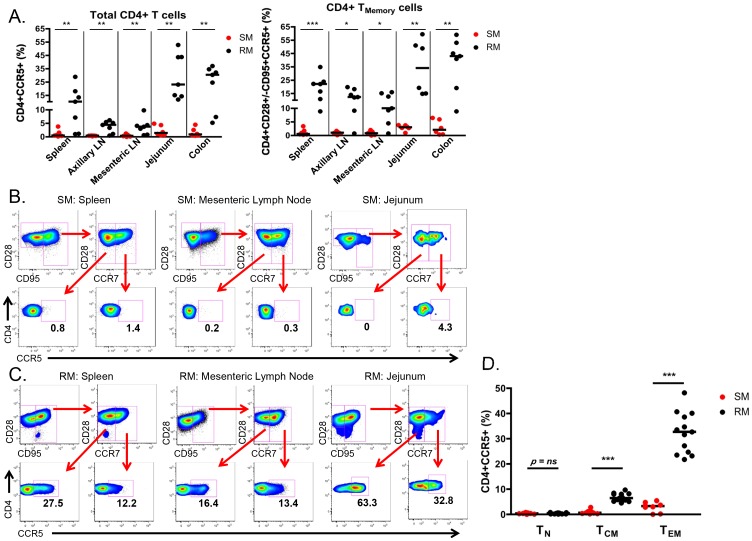
Few CD4+CCR5+ SIV target cells present in infant SMs. (A) The proportion of CD4+CCR5+ SIV target cells was measured by flow cytometry in total and memory CD4+ T cells isolated from lymphoid and gastrointestinal tissues of infant SMs (red) and RMs (black) at necropsy. Representative flow plots showing CCR5 expression on effector memory (CD28+CD95+CCR7−) and central memory (CD28+CD95+CCR7+) CD4+ T cells from the spleen, mesenteric LN, and jejunum from infant SMs (B) and RMs (C). (D) The percentage of CD4+CCR5+ T cells among the naïve (CD28+CD95−CCR7+), central memory (CD28+/−CD95+CCR7+), and effector memory (CD28+/−CD95+CCR7−) subsets in the peripheral blood of infant SMs (red) and RMs (black). Mann-Whitney test was used to determine significance. * p<0.05; ** p<0.01; *** p<0.001.

To better identify and quantitate SIV targets in infant SMs and RMs, we then determined the frequency of CD4+ T cells expressing CCR5 in blood, lymphoid tissues and the gastrointestinal tract. As shown in Fig. 5A, we found minimal expression of CCR5 on total and CD28+/−CD95+ memory CD4+ T cells in SM infants (median of <4% for all tissues). By comparison, RM infants had statistically significantly higher levels of CCR5+ total and memory CD4+ T cells at all sites. The difference was most pronounced in the RM spleen (median of 22.1% CCR5+CD4+ memory T cells), jejunum (median of 34% CCR5+CD4+ memory T cells), and colon (median of 43% CCR5+CD4+ memory T cells). Due to the small numbers of cells obtained from some tissues, we were not able to further distinguish the central and effector memory populations to assess their levels of CCR5 in all animals. When sufficient cell numbers could be analyzed (primarily in the lymph nodes and spleen, but also in the gastrointestinal tract of several animals), we consistently noted very low levels of CCR5 on central and effector memory subsets of SMs (Fig. 5B), but higher levels in RMs (Fig. 5C).

Finally, we performed a direct comparison of the levels of SIV target cells within T cell memory subsets found in the peripheral blood of infant SMs and RMs. Among circulating CD28+CD95−CCR7+ naïve CD4+ T cells the levels of CCR5 were low and not different between RMs and SMs (Fig 5D). However, we found significantly higher levels of CCR5 on CD28+CD95+CCR7+ central memory CD4+ T cells from RMs (median 6.4%, range 4.5–9.7%) compared to SMs (median 0.6%, range 0.2–2.8%; p<0.001). CCR5 was also expressed by a greater percentage of CD28+/−CD95+CCR7− effector memory CD4+ T cells from RMs (median 32.7%, range 21.8–48.2%) compared to SMs (median 3.3%, range 0–5.4%; p<0.001). These data confirm that infant RMs have ample target cells for SIV infection [29]. In summary, the level of memory CD4+CCR5+ T cells that are preferential targets for SIV infection is significantly lower in SM infants as compared to RM infants.

### Differential upregulation of CCR5 with age in infant RMs vs. SMs in oral mucosa, esophagus, and tonsils

**Figure 6:**
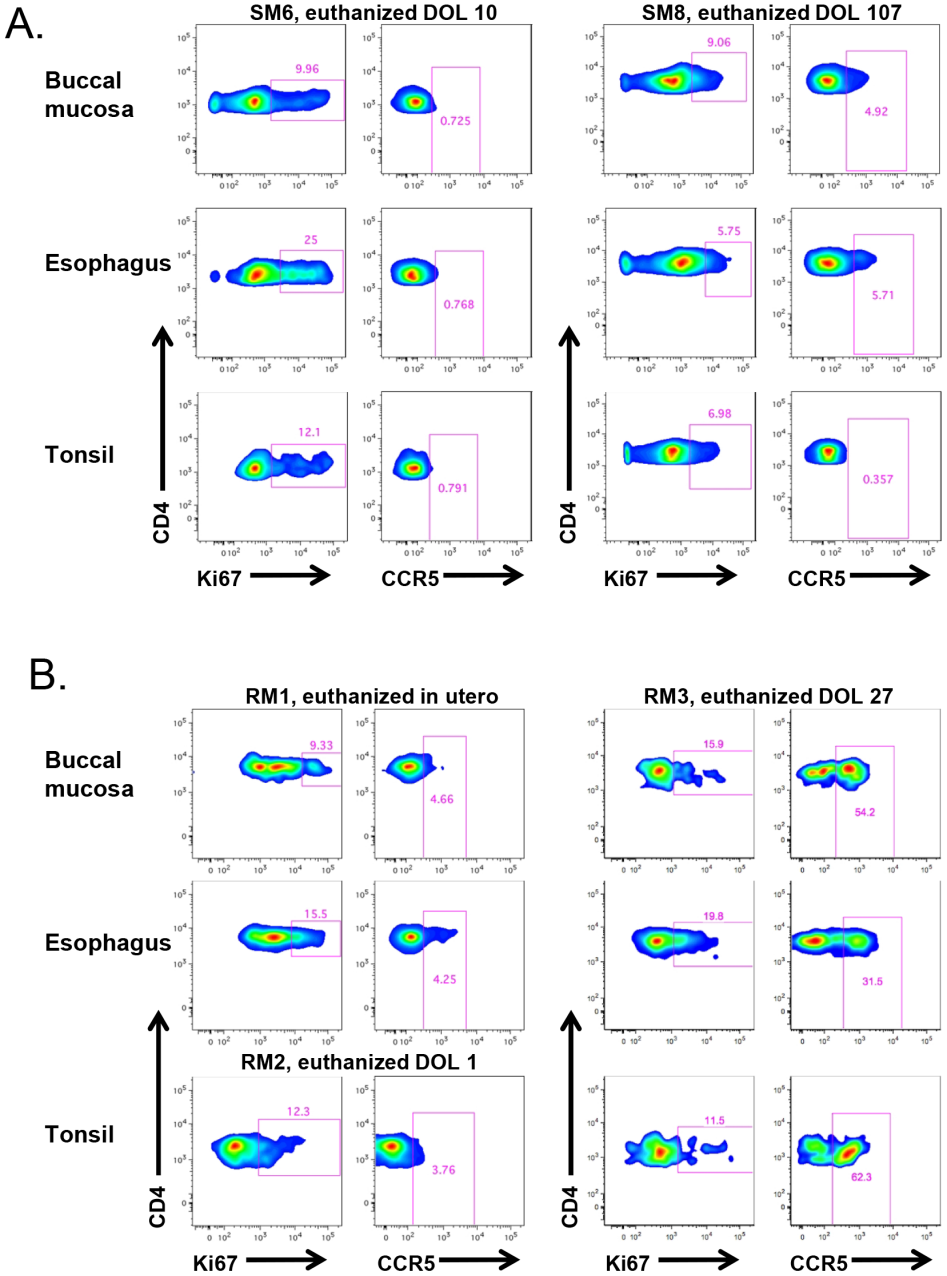
RM, but not SM, infants upregulate CCR5 expression on oral and upper gastrointestinal tract tissues early in life. The level of proliferation (Ki-67+) and SIV coreceptor expression (CCR5+) on CD4+ T cells isolated from the buccal mucosa, esophagus, and tonsils of representative SM (A) and RM (B) infants. CCR5 levels on CD4+ T cells remain low at these sites in SMs beyond three months of age, but are elevated before one month of age in RMs. DOL  =  day of life.

We next sought to assess SIV target cells at sites that are the first to encounter HIV/SIV transmitted through breast milk, specifically the buccal mucosa, tonsils, and esophagus. Figure 6A shows representative data from two SM infants, one who was euthanized on the 10th day of life and another who was euthanized at 107 days. We found a similar pattern of robust proliferation with little CCR5 expression on CD4+ T cells from these sites, although in the older infant the level of CCR5 was slightly increased in the gastrointestinal tissues (4.9–5.7% of CD4+ T cells), but not in tonsillar tissue (Fig. 6A). In Figure 6B, we present corresponding data from RM infants. Interestingly, we found low levels of CCR5 on CD4+ T cells in the buccal mucosa, esophagus, and tonsil in an RM infant euthanized in utero (secondary to maternal complications) and on the first day of life. However, we observed a massive expansion of CD4+CCR5+ T cells (31–62%) in all three sites before one month of age, clearly indicating that high levels of SIV target cells are present in RM infants early in life.

### The alternative SIV coreceptors GPR15 and CXCR6 are not highly expressed in SM infants

**Figure 7:**
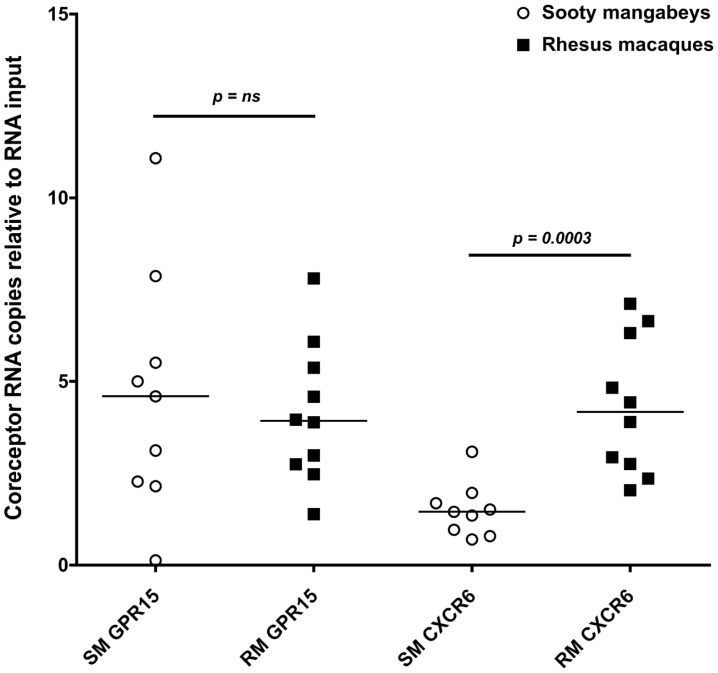
Alternative coreceptor expression in infants SMs and RMs. RNA expression of the alternative coreceptors GPR15 and CXCR6 was measured in sorted CD4+ T cells from infant SMs and RMs by real time PCR. CD4+ T cells were negatively selected by MACS microbeads and purity was assessed by flow cytometry (>80% for all animals). Mann Whitney test was used to assess significance.

Due to a concern that coreceptors other than CCR5 have the potential to mediate SIV infection of SM infants, we measured the expression of CXCR6 and GPR15 by real time PCR in sorted CD4+ T cells from infant SMs and RMs. These coreceptors have previously been demonstrated to mediate SIVsmm entry in transfected cell lines and may be involved in SIV infection of SMs with CCR5 deletions [30], [31]. As shown in Figure 7, we found very low levels of these coreceptors in the SM infants (whose parents all had wild type CCR5 genotypes). In addition, we measured cell surface GPR15 by flow cytometry on infant SM CD4+ T cells both pre- and post-activation with Concanavalin A and IL-2 and did not find significant expression (data not shown). CXCR6 staining was not performed due to lack of commercially available monoclonal antiboides that cross-react with SMs and the minimal RNA expression we observed (Fig. 7). In summary, these data show that SM infants, unlike RM infants, have limited availability of SIV target cells in multiple tissues, thus providing a potential mechanism of resistance from MTIT in this natural host species.

## Discussion

Mother to child transmission of HIV still accounts for approximately 370,000 new pediatric infections per year, with close to 50% of infections attributed to breastfeeding [1]. Despite similar plasma viremia and breastfeeding practices (e.g., frequency and duration), natural hosts and non-natural hosts for SIV demonstrate highly divergent rates of MTIT in the wild and in captivity [4]–[8], [32], [33]. We have previously shown that the rate of MTIT of SIV in the natural host sooty mangabey (SM) is significantly lower than in the non-natural host rhesus macaque (RM) and, critically, is also significantly lower than the rate of MTCT of HIV in the absence of any preventative intervention [3]. In this study, we investigated the physiologic mechanisms underlying the rarity of MTIT in SIV-infected SMs.

We examined three potential mechanisms to explain the low rates of MTIT of SIV in SMs. The first potential mechanism is that breast milk of natural hosts is less infectious because of lower viral loads. It should be noted that this study represents the first ever analysis of breast milk from SMs. The evidence did not support this hypothesis, as we have shown here that SMs and RMs have similar levels of cell-free SIV RNA and cell-associated SIV DNA in breast milk over a 5-month period, with SMs actually demonstrating more consistent virus shedding into the breast milk compartment (Fig. 1). Similar milk viral loads were reported in a comparison of RMs with another natural host species, the African green monkey, from a single sampling day [11]. Thus, the resistance to MTIT in natural hosts does not appear to be related to the inability of virus to traverse the mammary epithelium or replicate locally within the breast.

The second potential mechanism to account for restricted MTIT in SMs is that the breast milk of natural hosts contains inhibitory factors that limit virus infectivity at the level of infant mucosa. There have been a number of innate factors described in human milk that either inhibit HIV infection in vitro or that have been epidemiologically linked to lower rates of transmission, such as lactoferrin, long chain polyunsaturated fatty acids, oligosaccharides, SLP1, and a number of cytokines/chemokines [21]. We hypothesized that SMs might possess a specific factor (or factors) that interferes with SIV infection, perhaps similar to the VIRIP isolated from human blood [23]. However, while we found that milk from both SMs and RMs partially inhibits infection of Tzm-bl cells at the 1∶10 and 1∶50 dilutions, SMs did not more efficiently block infection (Fig. 3). When specific cytokines and chemokines were quantified in milk from both species, SMs consistently expressed more of the pro-inflammatory chemokine IL-8 than RMs (Table 3). IL-8 is increased in cases of subclinical mastitis [34] and milk containing high-level IL-8 (in addition to other pro-inflammatory cytokines) has been shown to enhance cell-associated HIV infection [35], a finding that is difficult to reconcile with protection from MTIT in SMs. In addition, levels of specific factors with known antiviral activity, such as IFN-β, IFN-γ, TNF-α, MIP-1α, MIP-1β, RANTES, and MDC, did not differ between the natural and non-natural hosts for SIV (Table 3 and data not shown).

The third mechanism we investigated is that infant SMs have limited numbers of CD4+CCR5+ target cells for SIV infection. We favored this hypothesis as we have previously shown that adult SMs express lower levels of CCR5 on CD4+ T cells compared to RMs, and particularly so in central memory CD4+ T cells [24], [36]. However, the role of CCR5 expression on infant CD4+ T cells as a mechanism to prevent MTIT of SIV in SMs had not been previously investigated. In recent work, intra-rectal SIV infection of African green monkeys and pigtailed macaques was found to be dependent on the level of CD4+CCR5+ T cells at the site of exposure, with a significant positive correlation between the percentage of CD4+CCR5+ T cells and the number of transmitted founder viruses [9]. Moreover, this study reported low levels of CCR5+CD4+ T cells in juvenile African green monkeys, with an increased percentage of CCR5+CD4+ T cells found in the blood and jejunum of sexually mature animals. In the present study, we greatly expanded the number of mucosal and lymphoid sites interrogated in a natural host model, examined CCR5 expression in CD4+ T cell subsets, and provided the first ever characterization of SIV target cells in infant SMs. Consistent with the hypothesis that MTIT of SIV is dictated by the level of CD4+CCR5+ target cells, we found extremely low percentages of these cells at all sites in infant SMs, including those that are in first contact with ingested virus such as the oral and esophageal mucosa (Fig. 6). In contrast, infant RMs had a much higher percentage of circulating CD4+CCR5+ T cells within both the central and effector memory subsets (Fig. 5D) as well as higher levels of CD4+CCR5+ memory T cells in all examined tissues, which may explain their increased susceptibility to MTIT of SIV compared to natural hosts.

Interestingly, while cord blood CD4+ T cells do not express CCR5 in humans, an abundant presence of CD4+CCR5+ memory T cells has been found in the gastrointestinal tract of human infants and fetal tissue [37]. These cells were highly susceptible to HIV infection in vitro and probably represent the main targets for infection following ingestion of HIV in breast milk. We observed lower levels of SIV target cells in the oral mucosa, esophagus, and tonsils of RMs euthanized in utero and on the first day of life compared to an RM euthanized at 27 days of life (Fig. 6), suggesting rapid upregulation of CCR5 expression post-partum in this non-natural host. This increase in CCR5 expression correlates with high rates of breastfeeding transmission of SIV in RMs [4], [6]. In this context, the major biological feature that may explain the differential rates of SIV MTIT in natural hosts as compared to non-natural host RMs or humans is the availability of target cells at mucosal and lymphoid sites.

We have long known that SIV-infected SMs maintain high viral loads and, in chronic infection, that CD4+ effector memory T cells that express higher levels of CCR5 (compared to central memory T cells) likely produce the majority of virus [24]. SIV infection of a small percentage of sooty mangabeys homozygous for a deletion mutation in CCR5 (delta 2 or delta 24) that abrogates its cell surface expression may be mediated by the alternative coreceptors GPR15 and CXCR6 [38]. Interestingly, viral loads in these CCR5 knock-out SMs are approximately 0.5 to 1-log lower than in animals wild type for CCR5. We here investigated the potential role of the alternative SIV coreceptors GPR15 and CXCR6 in mediating SIV transmission to SM infants and found their expression to be very low in CD4+ T cells (Fig. 7), indicating that SIV infection of target cells is unlikely to be mediated by these pathways in infant SMs.

One limitation of this study is that, due to federal regulations, we could only perform opportunistic collection of tissues from euthanized SM infants, and several of our study animals died from sepsis-related causes that might have influenced the overall level of CD4+ T cell activation. In light of this limitation, however, the very low level of CCR5 we observed in SM infants is perhaps even more striking. A second limitation is that we did not specifically investigate the role of the adaptive immune response, present either in milk or in the circulation of SM infants, as a potential mechanistic factor that limits MTIT of SIV. The rationale to prioritize other virological and immunological analyses in the samples that were available from the animals included in this study was that (i) SIV-infected SMs show circulating virus-specific CD8+ T cell responses that are of modest magnitude and are ineffective in controlling virus replication as well as low in vitro titers of autologous neutralizing antibodies in the plasma [39], [40], and (ii) the robust levels of virus, both cell-free and cell-associated, in the milk of lactating SIV-infected SMs that argue against immune control. While a neutralization response against the challenge virus was detected in both plasma and milk from SIV-infected African green monkeys, the importance of this finding remains unclear given the robust viral loads (10^4^–10^5^ copies / ml plasma) observed in both body fluids [11].

Interestingly, in the rare SMs who become infected via vertical transmission as well as experimentally-infected neonatal African green monkeys, viral loads are 1- to 2-log lower than those seen in animals who become infected later in life by the horizontal route [3], [41]. This low-level viremia is also consistent with the limited availability of SIV target cells in natural host infants and supports the hypothesis that low expression of CCR5 on CD4+ T cells has evolved as a major mechanism of protection from vertical transmission of SIV. The limited expression of CCR5 in natural hosts differs from what is seen in human infants (as described above) and it should be remembered that perinatally HIV-infected infants maintain high viral loads in absence of therapy and rapidly progress to AIDS. One could speculate that, thousands of years ago, natural host infants displayed a similar phenotype to perinatally HIV-infected children, resulting in poor clinical outcomes and “dead-end” infection in terms of transmission. Coevolution of virus and host may have selected for limited MTIT by decreased expression of CCR5 on all CD4+ T cell subsets in infants with widespread infection of older animals due to moderate expression of CCR5 on effector memory T cells. The consequent lack of pathogenesis in adult mangabeys is attributed to sparing of the central memory compartment from SIV infection [24], but it should be noted that the exact mechanism that determines cell surface expression of CCR5 on different cell subsets in SMs remains unknown. In this scenario, infants are spared a highly pathogenic and universally fatal infection and thus propagation of the species continues. At the same time, the virus is able replicate for many years in adult natural hosts and is readily horizontally transmitted. Efforts to harness this mode of protection from MTIT in natural hosts for therapeutic purposes may have a major impact on HIV prevention, treatment, and cure strategies.

## Materials and Methods

### Ethics statement

This study was conducted in strict accordance with USDA regulations and the recommendations in the Guide for the Care and Use of Laboratory Animals of the National Institutes of Health, and were approved by the Emory University Institutional Animal Care and Use Committee (AWA# A3180-01). SIV-infected animals were housed in standard non-human primate cages, received standard primate feed as well as fresh fruit and enrichment daily, and had continual access to water. Cages also contained additional sources of animal enrichment including objects such as perching and other manipulanda. Animal welfare was monitored daily. Appropriate procedures were performed to ensure that potential distress, pain, or discomfort was alleviated. The sedatives Ketamine (10 mg/kg) or Telazol (4 mg/kg) were used for blood draws and expression of breast milk. Euthanasia of infant SMs or RMs, using Pentobarbital (100 mg/kg) under anesthesia, was performed only when deemed clinically necessary by veterinary medical staff and according to IACUC endpoint guidelines.

### Animals

For the studies of induced lactation, three chronically SIV-infected SMs (*Cercocebus atys*) and RMs (*Macaca mulatta*) were followed. As part of earlier studies conducted prior to 2006 [42], [43], SMs were infected intravenously with 1 ml of plasma obtained from a naturally SIVsmm-infected SM (viral load 4×10^6^ copies / ml plasma). RMs were infected intravenously with 200 TCID_50_ SIVmac239 and were Mamu-B*08 and –B*17 negative. Milk was also obtained from SIV-uninfected SMs and RMs in breeding colonies. For the studies of SIV target cells in tissues, SM and RM infants who died within the first year of life underwent necropsy (Table 4). For the measurement of SIV target cells in CD4+ T cell memory subsets in peripheral blood, SMs and RMs <24 months of age were used. All RM and SM infants were all born to SIV-uninfected parents. All parents of the SM infants studied were homozygous wild type for the CCR5 gene. All animals were housed at the Yerkes National Primate Research Center.

### Pharmacologic induction of lactation

Nonpregnant female SMs and RMs were given depot medroxyprogesterone (3 mg/kg) and increasing doses of estradiol (0.1–0.25 mg/kg) intramuscularly to induce mammary gland maturation. Endogenous prolactin production was stimulated by oral administration of the dopamine antagonist haloperidol (0.25–0.45 mg/kg twice daily). Milk was collected three times per week via manual expression following intramuscular injection of 4–10 units of oxytocin. Doses were optimized for maximum milk collection and modeled after [10], [11]. Serum levels of progesterone, estradiol, and prolactin were monitored by ELISA (ALPCO Diagnostics). Levels of mononuclear cells in peripheral blood, including T cell subsets, as well as their proliferation, activation, and SIV coreceptor expression were measured by flow cytometry throughout the period of induced lactation.

### Breast milk processing

Milk was separated into the lipid layer, de-fatted (“skim”) milk, and cellular portion by centrifugation at 710×g for 20 min. The cell pellet was washed three times prior to staining for flow cytometry or freezing in RLT plus (Qiagen) for subsequent viral quantification.

### Tissue processing

Peripheral blood mononuclear cells (PBMCs) were isolated by gradient centrifugation. Procedures for processing of lymphoid and gastrointestinal tissues and isolation of lymphocytes were performed as described in [28]. Tonsils were processed in the same fashion as lymph nodes. Buccal mucosa was processed similar to other gastrointestinal sites.

### Phenotyping of PBMC, breast milk cells, and tissue-derived cells

Immunophenotyping was performed according to standard procedures using multicolor flow cytometry and monoclonal antibodies that were originally designed for humans or macaques and have been found to be cross-reactive in SMs. For analysis of SIV target cells in infants the following antibodies were used: Live/Dead Fixable Aqua from Invitrogen; CD4-Pacific Blue (OKT4) from BioLegend; CD28-PE-Texas Red (CD28.2) from Beckman-Coulter; CD95-PE-Cy5 (DX2) from eBioscience; CD8-APC-Cy7 (SK1), CD3-Alexa 700 (SP34-2), CCR7-PE-Cy7 (3D12), Ki-67-FITC (B56), CCR5-PE (3A9) all from Becton Dickinson. For analysis of RM and SM PBMCs the following additional antibodies were used: CD62L-PE (SK11), CCR5-APC (3A9), CD14-Pacific Blue (M5E2), CD11c-APC (S-HCL-3), HLA-DR-APC-Cy7 (G46-6/L243) all from Becton Dickinson; CD95-PE-Cy7 (DX2) and CD123-PE-Cy7 (6H6) from eBioscience; CD20-PE-TexasRed (B9E9) from Beckman-Coulter. For analysis of RM and SM breast milk cells, the following additional antibodies were used: CD45-V450 (D058-1283), CD28-PE-Cy5 (CD28.2), CD3-PerCP-Cy5.5 (SP34-2), CD14-FITC (MphageP9), Ki-67-Alexa 700 (B56) all from Becton Dickinson; CD8-Pacific Orange (3B5) and CD4-PE-Cy5.5 (S3.5) from Invitrogen. Flow cytometric acquisition was performed on an LSRII cytometer driven by the FACS DiVa software (Becton Dickenson). Analysis of the acquired data was performed using FlowJo software (TreeStar).

### SIVmac239 and SIVsmm RNA and DNA quantitative RT-PCR

The QIAamp viral RNA kit (Qiagen) was used to extract viral RNA from SM and RM skim milk and SM plasma, according to the manufacturer's recommendations. RM plasma was extracted with the Magna Pure LC (Roche Diagnostics; Indianapolis, IN), according to the manufacturer's recommendations. Plasma viral RNA quantification was performed as previously described [26]. RM skim milk RNA was quantified using SIVgag primers and probe along with conditions described in [44] with a sensitivity of 153 copies/ml. SM skim milk RNA was quantified with primers and probe along with conditions described in [45] with slight modification: 27 µl of RNA were loaded into a 100 µl two-step reaction with a sensitivity of 53 copies/ml. Skim milk spiked with known quantities of either SIVmac239 or SIVsmm were used as positive controls. Samples with undetectable SIV RNA were assigned a level of half of the lower limit of detection for graphical purposes.

Cell-associated proviral DNA from SM and RM breast milk cells and PBMC was obtained by extracting total DNA with the AllPrep DNA/RNA kit (Qiagen). Then, 80 ng per 15 µl of DNA were loaded into a 50 µl reaction using an SIVgag primer/probe set (RM) or an SIVutr primer/probe set (SM) as described in [44], [45]. Albumin was used as an internal control to quantify cell number against an external standard curve. Albumin primers and probe along with qPCR conditions were previously described in [46]. The proportion of SIV+ cells was determined as previously reported in [11]. The sensitivity of the assay is 5 SIV DNA copies per 10^5^ cells. Samples with undetectable SIV DNA were assigned a level of half of the lower limit of detection for graphical purposes.

### Levels of cytokines and chemokines

Plasma and milk levels of twenty-eight cytokines, chemokines, and growth factors were measured using a sandwich immunoassay-based protein array system, the Monkey Magnetic 28-Plex Panel (Invitrogen) as instructed by the manufacturer and then read by the Luminex 100 reader (Luminex Corp), which uses fluorescent bead-based technology. IFN-β in milk was measured using a simian ELISA kit (Ucsn Life Sciences Inc.)

### In vitro SIV infection

Virions, pseudotyped with one of four SIV envelopes (FFv18NOV04PLENV2.1, FWk12AUG04ENV4.1, SIVmac251.6, or SIVmac251.CS), were created via co-transfection with an Env-deficient subtype B HIV-1 proviral plasmid (pSG3Δenv) in 293T cells using FuGENE HD (Promega). Viral titers were determined via infection and β-galactosidase staining of the Tzm-bl indicator cell line. Five-fold serial dilutions (beginning at 1∶10) of de-fatted milk from RMs (8 animals) or SMs (7 animals) were then tested for inhibitory potential against these viral pseudotypes. Roughly 6,000 Tzm-bl cells per well were plated and cultured overnight in flat-bottomed 96-well plates. Pseudovirus (2,000 IU) in DMEM with ∼3.5% FBS (HyClone), 40 µg/ml DEAE-dextran was incubated with diluted milk for approximately 1 hour before 100 µl of this mixture were added to plated Tzm-bl cells for a 48 hr infection. Cells were subsequently lysed and evaluated for luciferase activity, and raw data was retrieved from a BioTek Synergy HT multi-mode microplate reader with Gen 5, v2.00 software. The average background luminescence from a set of uninfected wells was subtracted from each experimental well, infectivity curves were generated using GraphPad Prism v5.0d where values from experimental wells were compared against a well containing virus only with no milk, and IC_50_ values were determined using linear regression in Microsoft Excel for Mac 2011, v14.2.3.

### SIV coreceptor RNA quantification

CD4+ T cells were negatively selected from frozen PBMC using magnetically labeled microbeads and subsequent column purification according to the manufacturer's protocol (Miltenyi Biotec). The purity of enriched CD4+ T cells was evaluated by flow cytometry and was >80% in all cases. Sorted CD4+ T cells were lysed in RLT buffer (QIAGEN) and stored at −80°C. Total RNA from PBMC was purified using RNeasy mini kit (QIAGEN) according to manufacturer's protocol utilizing on-column DNAse digestion. RNA quantity was measured using Nanodrop analysis. RNA samples (0.3–1.0 µg) were reverse transcribed in a volume of 20 µl using random hexamers and the Super Script II kit (Invitrogen) as previously described [47] and 0.2 µl of cDNA was used for real time SYBR green PCR analysis using an ABI 7900 HT instrument (Applied Biosystems). Primer sequences for PCR were CXCR6: Fwd 5′-ACCCTGTGCTCTATGCCTTTGTCA-3′, Rev 5′-AAGGGAGACAGCCAATGTCCTTCA-3′; GPR15: Fwd 5′-ACTGCAGTGTCTTCCTGCTCACTT-3′, Rev 5′-AAACCAGATGCTGGCGCAAACTAC-3′. Serial dilutions of pcDNA3.1-smGPR15, and CXCR6 plasmids (kindly provided by R. Collman, [30], [47]) were used for the quantification. Results were normalized to input RNA.

### Statistical analyses

Comparisons between the level of SIV target cells in RMs and SMs as well as the level of cytokines and chemokines in milk of RMs and SMs were made using the nonparametric Mann-Whitney test. The inhibitory capacities of RM and SM milk against in vitro SIV infection were compared at the 1∶10 and 1∶50 dilutions by nonparametric Mann-Whitney test. Comparisons between cell-free and cell-associated virus levels in blood and milk from RMs vs. SMs over time were made using area under the curve analyses followed by nonparametric Mann-Whitney test. In all cases, significance was attributed at p<0.05. All analyses were conducted using GraphPad Prism 4.0c or 5.0d.
